# Modified 5-Item Frailty Index Is Associated with Postoperative Delirium but Not Survival in Patients Undergoing Surgery for Oral Squamous Cell Carcinoma

**DOI:** 10.3390/diagnostics16162551

**Published:** 2026-08-13

**Authors:** Kenji Yamagata, Satoshi Fukuzawa, Shohei Takaoka, Jumpei Shirakawa, Kie Nakatani, Eri Sasabe, Yukio Yoshioka, Naomi Kanno, Fumihiko Uchida

**Affiliations:** 1 Department of Oral and Maxillofacial Surgery, Kochi Medical School, Kochi University, 185-1 Kohasu, Oko-Cho, Nankoku 783-8505, Japan; juneosur0610@gmail.com (J.S.); tnakatani_kie@kochi-u.ac.jp (K.N.); yoshieri@kochi-u.ac.jp (E.S.); k73347145@kochi-u.ac.jp (Y.Y.); 2 Department of Oral and Maxillofacial Surgery, University of Tsukuba Hospital, Tsukuba 305-8576, Japan; sfkuzawa3104@yahoo.co.jp (S.F.); sho.tmdu@gmail.com (S.T.); nkanno@md.tsukuba.ac.jp (N.K.); f-uchida@md.tsukuba.ac.jp (F.U.)

**Keywords:** frailty, mFI-5, oral squamous cell carcinoma, postoperative complications, delirium, survival, head and neck cancer

## Abstract

**Background/Objectives:** Frailty is increasingly recognized as a clinically relevant marker of reduced physiological reserve in surgical oncology. The modified 5-item frailty index (mFI-5) is simple and practical, but its clinical significance in oral squamous cell carcinoma (OSCC) remains incompletely defined. This study evaluated the association between mFI-5-defined frailty and postoperative complications, especially postoperative delirium, as well as survival outcomes in patients undergoing surgery for OSCC. **Methods:** We retrospectively analyzed 127 patients who underwent surgical resection for OSCC with postoperative high care unit (HCU) management between 2013 and 2021. Frailty was defined as an mFI-5 score of ≥2. Clinical characteristics, postoperative outcomes, HCU stay, length of hospital stay, overall survival (OS), and disease-free survival (DFS) were compared between frail and non-frail groups. Univariable logistic regression was used to estimate odds ratios (ORs) and 95% confidence intervals (CIs) for postoperative complications. An exploratory multivariable logistic regression analysis for postoperative delirium was performed using age ≥65 years and sex as covariates. Delirium was retrospectively assessed from clinical documentation considered consistent with DSM-5 criteria. **Results:** Twenty-one patients (16.5%) were classified as frail. Postoperative delirium occurred more frequently in frail patients than in non-frail patients (42.9% vs. 19.8%; *p* = 0.023). In a multivariable logistic regression model adjusted for age ≥65 years and sex, mFI-5-defined frailty was significantly associated with postoperative delirium (adjusted OR, 3.07; 95% CI, 1.08–8.60; *p* = 0.035). No significant association was observed for pneumonia, surgical site infection, or free-flap reoperation. Frailty was not significantly associated with HCU stay, length of hospital stay, OS, or DFS. **Conclusions:** mFI-5-defined frailty was associated with postoperative delirium but not with survival outcomes in this OSCC cohort. Because this retrospective study was limited by sample size, comorbidity-driven mFI-5 scoring, non-standardized delirium screening, and potential residual confounding, mFI-5 should be interpreted as a convenient screening marker rather than a stand-alone predictor. Comprehensive perioperative assessment incorporating frailty, nutrition, sarcopenia, cognition, tumor burden, and treatment-related factors may better identify patients at risk.

## 1. Introduction

Frailty is a geriatric syndrome characterized by reduced physiological reserve, impaired homeostatic capacity, and increased vulnerability to external stressors. The concept was originally developed in older populations and has classically been described using phenotypic criteria such as weakness, exhaustion, slow walking speed, low physical activity, and unintentional weight loss [1,2]. In surgical oncology, frailty has become increasingly important because it may capture clinically relevant risk that is not fully reflected by chronological age, tumor stage, or conventional comorbidity scores. For patients with head and neck cancer (HNC), this issue is particularly relevant because major ablative surgery, neck dissection, airway management, prolonged anesthesia, and microvascular reconstruction can impose substantial physiological stress [3,4].

The modified 5-item frailty index (mFI-5), derived from the American College of Surgeons National Surgical Quality Improvement Program (ACS-NSQIP), is one of the most frequently used abbreviated frailty instruments in surgical studies [5]. It comprises diabetes mellitus, hypertension requiring medication, chronic obstructive pulmonary disease or pneumonia, congestive heart failure, and functional dependence. Its major advantages are simplicity, objectivity, and applicability to retrospective datasets. Recent studies and meta-analyses have shown that mFI-5 is associated with adverse postoperative outcomes in several surgical fields, including plastic and reconstructive surgery, otolaryngology, and head and neck reconstruction [6,7,8,9]. However, mFI-5 is not equivalent to the classic frailty phenotype. Most of its components reflect comorbidity burden rather than nutrition, sarcopenia, cognition, inflammation, mobility, or social vulnerability. Therefore, studies using mFI-5 should interpret findings as mFI-5-defined frailty, not as a complete assessment of biological frailty.

Oral squamous cell carcinoma (OSCC) is commonly treated by surgery, and advanced disease frequently requires composite resection, neck dissection, tracheostomy, and free-flap reconstruction. Postoperative complications in OSCC are multifactorial. Nutritional depletion, sarcopenia, advanced age, alcohol consumption, smoking history, long operative and anesthesia times, flap reconstruction, systemic inflammation, poor baseline function, cognitive vulnerability, and tumor stage may all influence postoperative morbidity [10,11,12,13,14,15,16,17,18,19]. Among postoperative complications, delirium is clinically important because it can delay rehabilitation, increase medical resource use, prolong hospital stay, impair quality of life, and potentially affect long-term functional recovery [10,12,14]. Recent evidence also suggests that preoperative cognitive impairment, malnutrition, sarcopenia, and frailty are closely related to postoperative delirium among patients undergoing cancer surgery [15,16,17].

Although frailty has been studied in HNC, data focusing on surgically treated OSCC remain limited. Moreover, the literature is inconsistent: some studies have reported significant associations between mFI-5 and complications or survival, whereas others have found inconsistent or weak predictive value, especially after considering procedure type and competing oncological factors [7,8,9,20,21,22,23,24]. This inconsistency raises an important clinical question: does mFI-5-defined frailty identify OSCC patients who are particularly vulnerable to postoperative complications, or do advanced tumor burden and treatment intensity dominate postoperative and survival outcomes? Therefore, the present study aimed to evaluate the association between mFI-5-defined frailty and postoperative complications, especially postoperative delirium, and to examine whether mFI-5-defined frailty was associated with OS and DFS in patients undergoing surgical treatment for OSCC.

## 2. Materials and Methods

### 2.1. Study Design and Patient Population

This retrospective cohort study included patients who underwent surgical resection for OSCC at the University of Tsukuba Hospital between January 2013 and December 2021. A total of 127 patients were included. The cohort consisted of 80 males and 47 females, with a median age of 68 years (range, 29–89 years). All patients received standardized perioperative management, including postoperative monitoring in a high-care unit (HCU), which was routinely used for patients undergoing major oral cancer surgery at our institution.

Patients were eligible if they had histologically confirmed OSCC and underwent curative-intent surgical resection with available perioperative and follow-up data. Patients were excluded if clinical records were incomplete, if postoperative follow-up data were insufficient for the evaluation of complications or survival outcomes, or if perioperative records did not allow reliable calculation of the mFI-5 score. Patients with recurrent disease, distant metastatic disease at the time of surgery, emergency surgery, or inadequate documentation of baseline status were not included. Because postoperative delirium was a primary focus, baseline neurocognitive disorders, psychiatric comorbidities, and pre-existing dementia or cognitive impairment were reviewed when documented in the medical record. However, standardized preoperative cognitive screening was not routinely performed throughout the study period. Perioperative exposures to sedatives, opioids, anticholinergic agents, and benzodiazepines were not consistently available in a structured format suitable for statistical adjustment. These issues were therefore considered important limitations.

This study was conducted in accordance with the Declaration of Helsinki and was approved by the institutional review board of the University of Tsukuba Hospital (No. R05-158; approved 31 October 2023). The requirement for informed consent was waived because of the retrospective nature of the study.

### 2.2. Frailty Assessment

Frailty was assessed using the mFI-5, a validated and widely used index derived from ACS-NSQIP [5]. The mFI-5 includes five variables: diabetes mellitus, hypertension requiring medication, chronic obstructive pulmonary disease or pneumonia, congestive heart failure, and functional dependence. Each item was assigned one point if present, and the total score ranged from 0 to 5.

For this retrospective study, each mFI-5 component was identified from the preoperative medical record using operational definitions consistent with the mFI-5 framework. Diabetes mellitus, chronic obstructive pulmonary disease or pneumonia, and congestive heart failure were considered present when documented as active preoperative diagnoses; hypertension was considered present when antihypertensive medication was required; and functional dependence was defined as requiring assistance with activities of daily living before surgery. Because these variables were abstracted from routine clinical documentation, disease-specific laboratory thresholds and severity grades were not uniformly available.

Patients with an mFI-5 score of ≥2 were classified as frail, whereas those with a score of <2 were classified as non-frail. This cutoff has been used in previous surgical studies to identify clinically meaningful frailty or increased perioperative vulnerability [6,7,8,9]. Nevertheless, because the mFI-5 is strongly driven by comorbid disease burden and contains only one functional domain, we used the term mFI-5-defined frailty throughout the manuscript to avoid overstating the construct measured by this index.

### 2.3. Data Collection and Variables

Clinical data were retrospectively extracted from electronic medical records. Patient-related variables included age, sex, smoking history, alcohol consumption, performance status (PS), and American Society of Anesthesiologists (ASA) classification. Tumor-related variables included the primary tumor site, tumor stage based on the Union for International Cancer Control (UICC) TNM classification, T classification, N classification, and M classification when available. In this cohort, no patient had documented distant metastasis at the time of surgery; therefore, all patients were considered M0 for the purposes of surgical treatment. Surgical variables included the type of reconstruction, free-flap use, and free-flap reoperation.

Postoperative outcomes evaluated in this study included pneumonia, postoperative delirium, surgical site infection (SSI), and reoperation. Postoperative delirium was retrospectively identified from clinical documentation considered consistent with the Diagnostic and Statistical Manual of Mental Disorders, Fifth Edition (DSM-5) criteria [25]. The assessment was based on documentation by attending physicians, surgeons, and nursing staff during postoperative ward and HCU care. Routine clinical observation included changes in attention, awareness, orientation, sleep–wake cycle, agitation, and abnormal behavior; however, standardized, scheduled delirium screening instruments, such as the Confusion Assessment Method (CAM), CAM-ICU, Intensive Care Delirium Screening Checklist (ICDSC), or Nursing Delirium Screening Scale (Nu-DESC), were not consistently used during the study period. Therefore, hypoactive delirium may have been under-recognized compared with studies using structured daily screening. Pre-existing dementia or cognitive impairment was recorded when described in the medical chart, but formal cognitive testing was not uniformly performed. Data on medication exposures that may influence delirium risk, including sedatives, opioids, anticholinergic agents, and benzodiazepines, could not be consistently extracted for adjusted analysis. SSI was defined according to the Centers for Disease Control and Prevention criteria as purulent drainage, wound dehiscence due to infection, abscess formation, or a diagnosis of infection by the attending surgeon [26]. Hospital course variables included the duration of HCU stay and the length of hospital stay. Survival outcomes were defined as overall survival (OS), calculated from the date of diagnosis to death from any cause, and disease-free survival (DFS), calculated from the date of diagnosis to recurrence or death.

Information on adjuvant therapy was not uniformly available for the entire cohort in a format that allowed reliable statistical analysis. Because postoperative radiotherapy, chemotherapy, and chemoradiotherapy can substantially affect survival and late morbidity, the absence of complete adjuvant treatment information was treated as a limitation when interpreting oncological outcomes.

### 2.4. Outcome Measures

The primary outcome was the association between mFI-5-defined frailty and postoperative complications, with particular emphasis on postoperative delirium. Secondary outcomes included postoperative pneumonia, SSI, free-flap reoperation, HCU stay, length of hospital stay, OS, and DFS. Because the primary analysis was exploratory and retrospective, the results were interpreted as associations rather than causal or independent predictive relationships.

### 2.5. Statistical Analysis

All statistical analyses were performed using JMP Student Edition version 18.2.2 (SAS Institute Inc., Cary, NC, USA). Categorical variables were compared using the chi-square test or Fisher’s exact test, as appropriate. Continuous variables were expressed as medians with ranges and compared using the Mann–Whitney U test. Survival analyses for OS and DFS were performed using the Kaplan–Meier method, and differences between groups were assessed using the log-rank test. All statistical tests were two-sided, and *p* < 0.05 was considered statistically significant.

To further clarify the statistical analysis, univariable logistic regression models were used to estimate odds ratios (ORs) and 95% confidence intervals (CIs) for the association between mFI-5-defined frailty and each postoperative complication. For postoperative delirium, a parsimonious multivariable logistic regression model adjusted for age ≥65 years and sex was performed because of the limited number of events. Adjusted ORs, 95% CIs, and *p*-values were reported in the Section 3. Multivariable analyses were not performed for pneumonia, SSI, or free-flap reoperation because of the small number of events. Therefore, these outcomes were presented as unadjusted regression-based associations, and the possibility of residual confounding was acknowledged as a limitation of the study.

## 3. Results

### 3.1. Patient Characteristics

A total of 127 patients were analyzed. Twenty-one patients (16.5%) were classified as frail based on an mFI-5 score of ≥2, and 106 patients (83.5%) were classified as non-frail. Baseline characteristics stratified by frailty status are summarized in Table 1. There were no statistically significant differences between the groups in age category, sex, smoking history, alcohol consumption, PS, ASA classification, primary tumor site, stage classification, T classification, N classification, reconstruction type, or free-flap reoperation.

Advanced disease was common in both groups. Stage IVA and IVB tumors accounted for most cases, and T4a disease was the most frequent T category. No patient had documented distant metastasis at the time of surgery. The predominance of advanced local disease was clinically important because tumor extent, operative complexity, and perioperative treatment intensity may influence both complications and survival outcomes independently of frailty status.

### 3.2. Postoperative Outcomes

Postoperative complications are shown in Table 1, Table 2 and Table 3. Postoperative delirium occurred more frequently in frail patients than in non-frail patients (9/21, 42.9% vs. 21/106, 19.8%; *p* = 0.023). Univariable logistic regression showed that mFI-5-defined frailty was associated with postoperative delirium (crude OR, 3.04; 95% CI, 1.11–8.16; *p* = 0.031). In the multivariable logistic regression model adjusted for age ≥65 years and sex, mFI-5-defined frailty was significantly associated with postoperative delirium (adjusted OR, 3.07; 95% CI, 1.08–8.60; *p* = 0.035). Neither age ≥65 years nor sex was significantly associated with postoperative delirium. Because the number of postoperative delirium events was limited, the model was restricted to a small number of clinically relevant covariates to reduce the risk of overfitting.

In contrast, univariable logistic regression did not show significant associations between mFI-5-defined frailty and postoperative pneumonia (OR, 1.01; 95% CI, 0.27–3.10), SSI (OR, 0.83; 95% CI, 0.12–3.36), or free-flap reoperation among patients who underwent free-flap reconstruction (OR, 3.48; 95% CI, 0.53–22.74). These results indicate that the observed association was most evident for delirium and was not consistently seen across all postoperative outcomes.

### 3.3. Distribution of mFI-5 Components

The distribution of mFI-5 components is shown in Table 4. Among patients with an mFI-5 score of 1, hypertension requiring medication was the most common component, followed by diabetes mellitus. Among patients with an mFI-5 score of 2, diabetes mellitus and hypertension were highly prevalent. Respiratory disease, congestive heart failure, and functional dependence were uncommon, and no patient demonstrated functional dependence. Thus, the mFI-5 profile in this cohort was driven mainly by metabolic and cardiovascular comorbidities rather than by functional impairment.

This pattern is important for interpretation. In this OSCC cohort, mFI-5-defined frailty may have represented comorbidity accumulation rather than a broader geriatric frailty phenotype. Consequently, the association with delirium may reflect the combined effects of vascular risk, metabolic disease, inflammatory vulnerability, and reduced resilience rather than frailty as measured by physical performance or nutritional status.

### 3.4. Hospital Course

**Figure 1:**
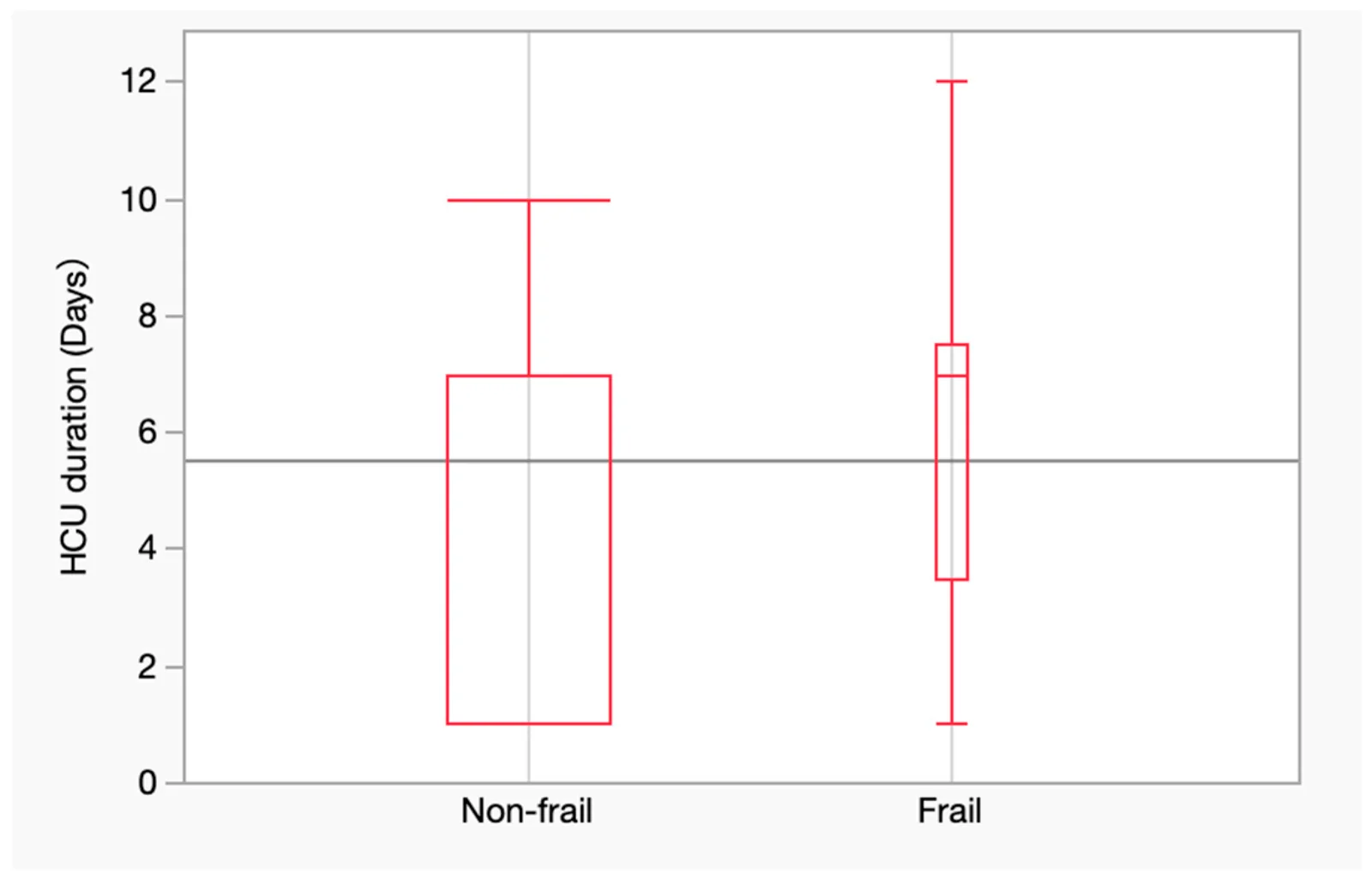
Association between high care unit (HCU) stay duration and frailty. The duration of HCU stay was compared between the frailty groups (*p* = 0.672).

**Figure 2:**
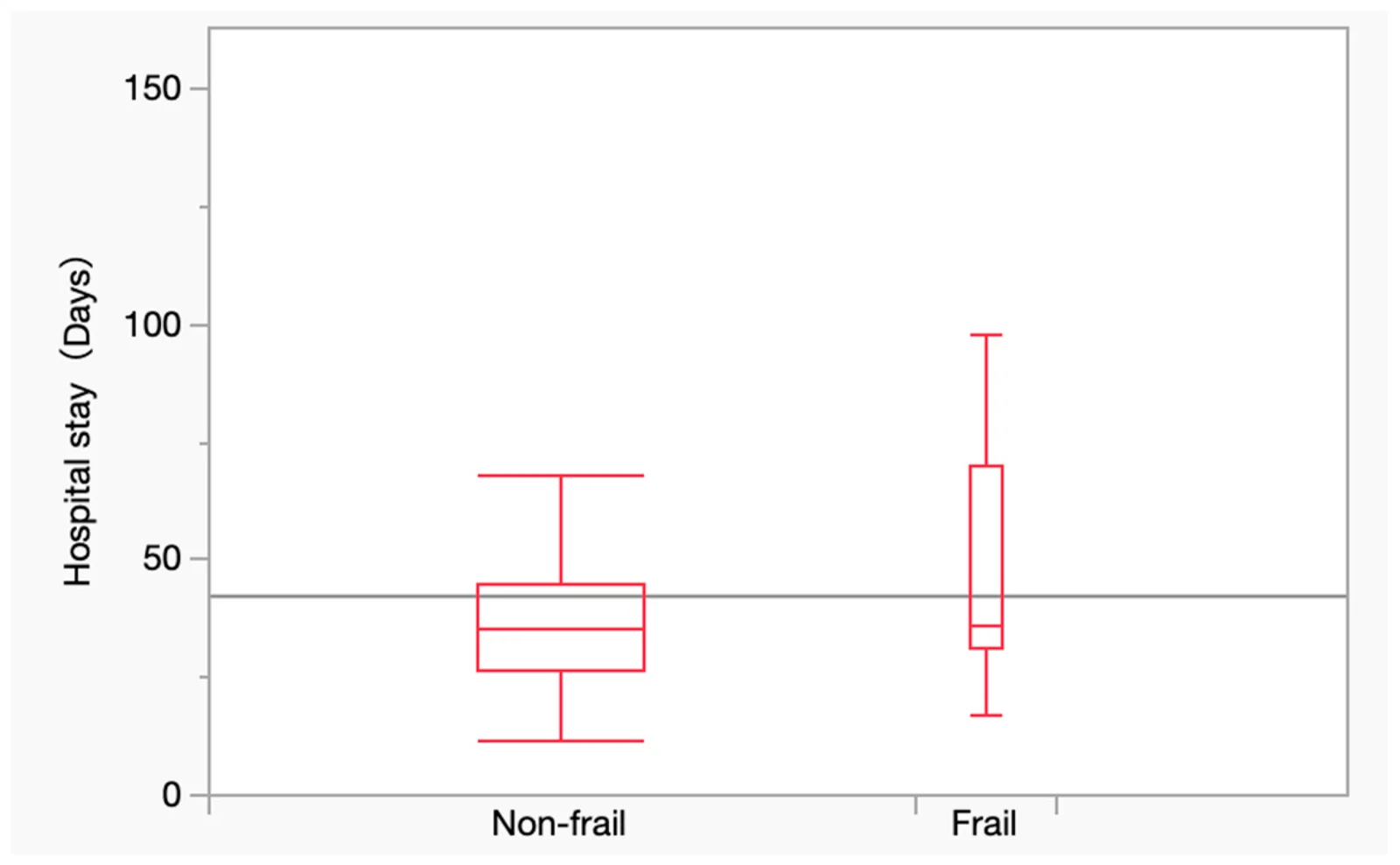
Association between length of hospital stay and frailty. The length of hospital stay was compared between the frailty groups (*p* = 0.085).

No significant differences were observed in the perioperative course between frail and non-frail patients. Median HCU stay was 7 days in both groups (*p* = 0.672; Figure 1). Length of hospital stay was also similar between groups, although there was a nonsignificant tendency toward a longer hospital stay in frail patients (median, 36 days vs. 35 days; *p* = 0.085; Figure 2). These findings suggest that mFI-5-defined frailty was not clearly associated with short-term resource utilization in this cohort, although the sample size may have limited the ability to detect small differences.

### 3.5. Survival Outcomes

**Figure 3:**
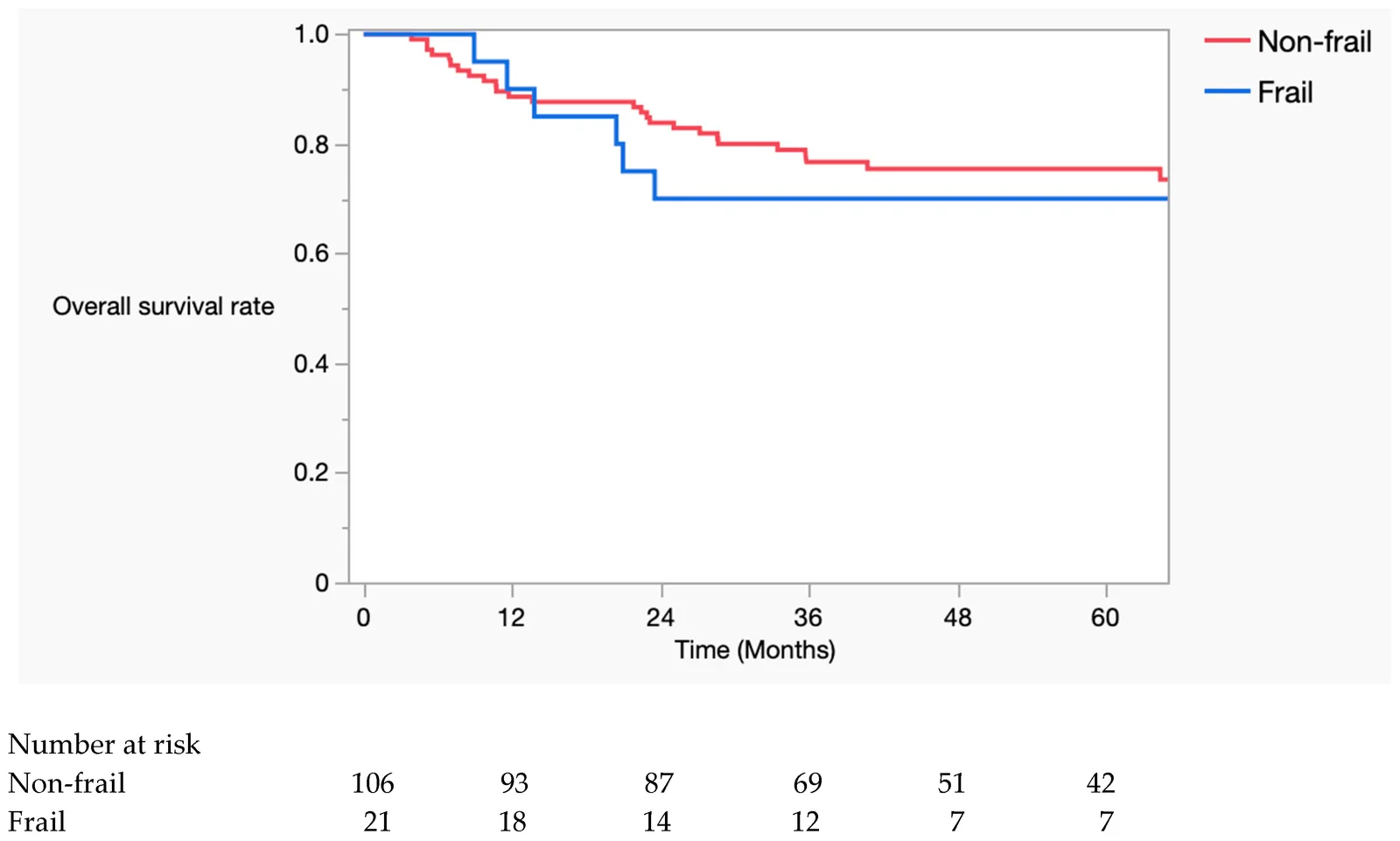
Association between overall survival (OS) and frailty. Kaplan–Meier analysis showed no significant difference in OS between the frailty groups (*p* = 0.628).

**Figure 4:**
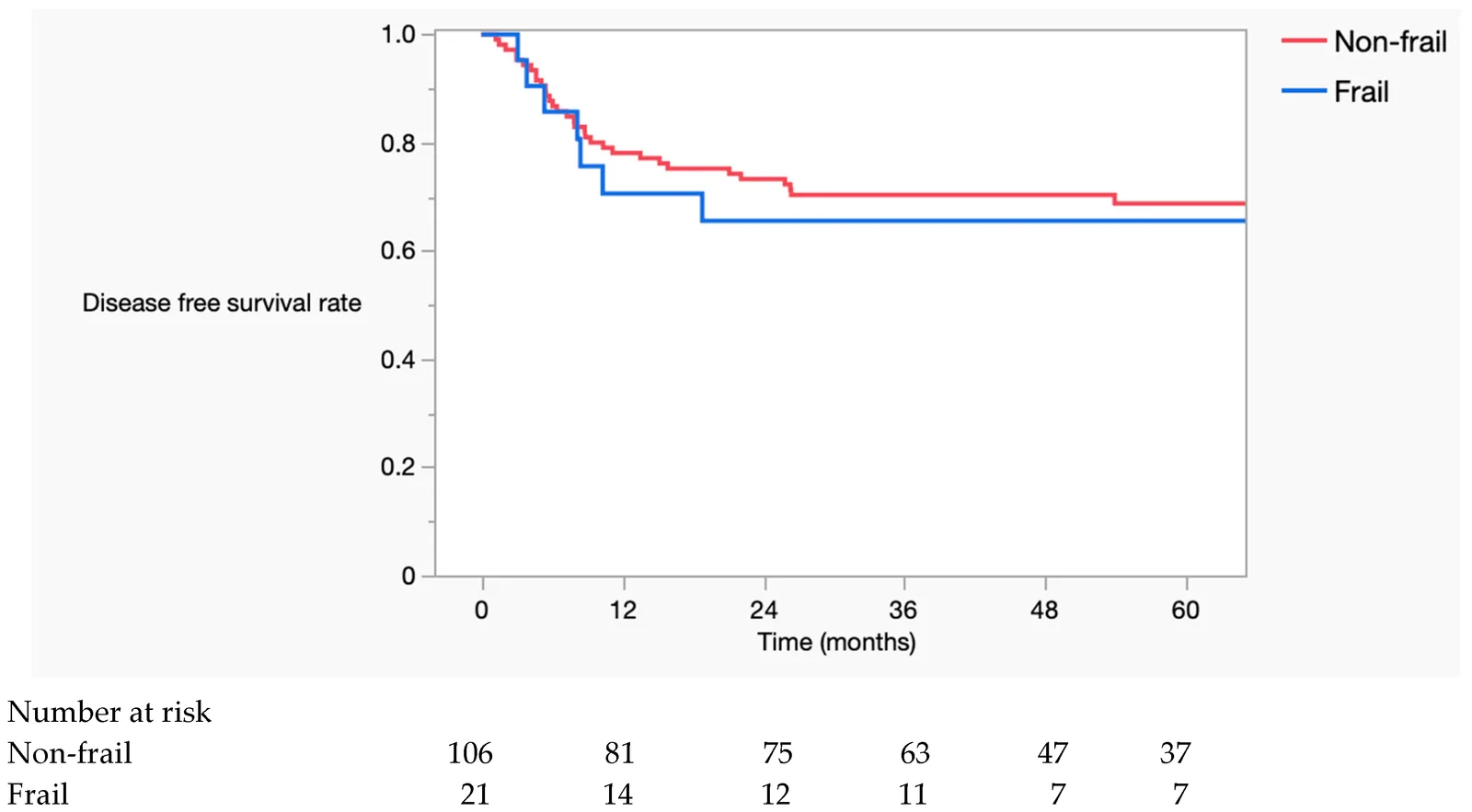
Association between disease-free survival (DFS) and frailty. Kaplan–Meier analysis demonstrated no significant difference in DFS between the frailty groups (*p* = 0.697).

Kaplan–Meier analyses showed no significant differences in OS or DFS between frail and non-frail groups. OS was comparable between the groups (log-rank *p* = 0.628; Figure 3), and DFS was also not significantly different (log-rank *p* = 0.697; Figure 4). The median follow-up duration was 44.2 months (range, 3.9–132.4 months). During follow-up, 32 patients died. For the OS analysis, 95 patients were censored at the last follow-up. For the DFS analysis, 39 patients experienced recurrence or death, and 88 patients were censored without an event. Recurrence patterns, which were not mutually exclusive, included local recurrence in 20 patients, regional recurrence in 15 patients, and distant metastasis in 23 patients. The 1-, 3-, and 5-year OS rates were 88.9%, 75.7%, and 74.6%, respectively, while the corresponding DFS rates were 76.9%, 69.5%, and 68.2%, respectively.

These results suggest that mFI-5-defined frailty was not associated with long-term oncological outcomes in this cohort. However, survival in OSCC is strongly affected by tumor stage, nodal disease, margin status, pathological risk factors, and adjuvant treatment. Therefore, the absence of a significant survival difference should not be interpreted as evidence that frailty has no oncological relevance; rather, it indicates that this study did not detect a survival association using the available data and unadjusted survival comparisons.

## 4. Discussion

The present study evaluated the clinical significance of mFI-5-defined frailty in patients undergoing surgery for OSCC. The main finding was that frailty, defined as an mFI-5 score of ≥2, was associated with a higher incidence of postoperative delirium in univariable logistic regression. This association remained significant in a multivariable logistic regression model adjusted for age ≥65 years and sex. In contrast, mFI-5-defined frailty was not significantly associated with pneumonia, SSI, free-flap reoperation, HCU stay, length of hospital stay, OS, or DFS. These findings suggest that mFI-5 may be useful as a simple preoperative screening marker for vulnerability to delirium, but the index should not be interpreted as a comprehensive or independent predictor of all postoperative and oncological outcomes.

The association between frailty and postoperative delirium is biologically plausible. Delirium is a multifactorial neuropsychiatric syndrome that arises from the interaction between predisposing vulnerability and perioperative precipitating factors. In major head and neck cancer surgery, risk factors include advanced age, male sex, smoking history, psychiatric history, higher ASA classification, low albumin level, postoperative insomnia, fluid imbalance, extensive surgical stress, and prolonged anesthesia [10,11,12,13]. Recent systematic reviews further emphasize the importance of preoperative cognitive function, sarcopenia, malnutrition, and frailty among patients undergoing cancer surgery [15,16,17]. The present findings are consistent with the broader literature and suggest that even a brief comorbidity-based frailty index may help identify patients with reduced resilience to perioperative stress.

Postoperative delirium has particular clinical relevance in OSCC. Patients may undergo prolonged surgical procedures, tracheostomy, complex wound care, enteral feeding, flap monitoring, and intensive postoperative rehabilitation. Delirium can interfere with communication, airway care, nutritional support, mobilization, and treatment adherence, thereby complicating recovery. Even if delirium does not directly affect OS or DFS, it may worsen functional recovery and quality of life. Therefore, identifying patients at increased risk is clinically meaningful. In practice, mFI-5 could be incorporated into a preoperative checklist to trigger preventive strategies such as medication review, sleep preservation, early mobilization, pain control, orientation protocols, family engagement, hydration and nutrition optimization, and early psychiatric consultation when appropriate.

A key point raised by the present analysis is that mFI-5-defined frailty was not associated with all complications. Pneumonia, SSI, and flap reoperation are influenced by multiple factors that are not fully captured by mFI-5, including oral hygiene, aspiration risk, operative time, blood loss, flap type, wound contamination, nutritional status, skeletal muscle mass, and postoperative care pathways. Recent OSCC studies have shown that sarcopenia and low skeletal muscle mass are associated with postoperative complications and survival, and that SSI may be influenced by muscle mass, comorbidities, and nutritional factors [18,19,27,28,29]. Therefore, a comorbidity-based index alone may be insufficient to predict wound-related or technical surgical outcomes. This may explain why delirium showed a clearer association with mFI-5 than SSI or reoperation in the present cohort.

The absence of a significant association between mFI-5-defined frailty and survival should also be interpreted carefully. In general, survival after OSCC surgery depends heavily on tumor biology, T and N categories, margin status, extranodal extension, adjuvant treatment, recurrence pattern, and treatment tolerance. In the present cohort, advanced-stage disease was common, and T4a tumors predominated in both frail and non-frail groups. This advanced tumor burden may have obscured the independent contribution of frailty to survival. Furthermore, the number of frail patients was small, and adjuvant therapy data were not uniformly available. Previous studies in laryngeal and oropharyngeal squamous cell carcinoma have suggested that frailty may be associated with worse survival in some settings [23,24], whereas other otolaryngology studies have demonstrated inconsistent predictive performance of mFI-5 when compared with alternative tools such as the Risk Analysis Index [22]. Therefore, the current results should be viewed as disease- and cohort-specific rather than definitive evidence. The limited sample size, particularly the 21 patients classified as frail, may also have reduced statistical power to detect a modest survival difference. Accordingly, the absence of a significant survival association in this cohort should be considered inconclusive rather than evidence of no effect, and larger multicenter cohorts are needed to clarify whether frailty modifies the impact of tumor burden on long-term outcomes.

Another important issue is the conceptual limitation of the mFI-5. The traditional frailty phenotype was developed as a geriatric construct involving physical performance and energy reserve [1]. By contrast, mFI-5 primarily captures five clinical conditions, four of which are comorbid diseases [5]. In this study, the frail group was largely defined by diabetes mellitus and hypertension, whereas functional dependence was absent. This means that mFI-5-defined frailty in our cohort may represent cardiometabolic comorbidity accumulation rather than multidimensional frailty. Accordingly, the term mFI-5-defined frailty is used throughout the manuscript to avoid implying that the index fully captures biological frailty. This distinction is clinically relevant because appropriate interventions differ: comorbidity optimization may reduce perioperative risk, but nutritional intervention, resistance training, cognitive assessment, and geriatric co-management may be needed to address multidimensional frailty.

Diabetes mellitus and hypertension were the predominant mFI-5 components in this cohort. These conditions may contribute to perioperative vulnerability through metabolic and vascular comorbidities; however, the mFI-5 records their presence rather than their severity or degree of control. Thus, patients with well-controlled disease and those with more severe disease receive the same component score, and the present data cannot determine whether diabetes or hypertension independently influenced OSCC-specific or survival outcomes. Their high prevalence should therefore be interpreted as an important contributor to the comorbidity burden captured by mFI-5 rather than as evidence of a direct oncological effect.

The study also highlights the need for more comprehensive risk stratification in OSCC surgery. Future models should integrate mFI-5 with age, PS, ASA class, nutritional markers such as albumin or the prognostic nutritional index, sarcopenia or skeletal muscle index, inflammatory markers, alcohol and smoking exposure, preoperative cognitive screening, operative duration, reconstruction type, tumor stage, and planned adjuvant therapy. Such a model may provide better discrimination than mFI-5 alone and may clarify whether frailty-related vulnerability remains significant across early and advanced disease strata. Stratification by tumor burden, such as early versus advanced disease based on T stage and nodal status, may be particularly useful because OSCC prognosis is strongly stage dependent [30].

This study has several limitations. First, it was retrospective and therefore subject to selection bias, information bias, and unmeasured confounding. Second, the sample size was modest, and only 21 patients were classified as frail. Although univariable logistic regression was performed to estimate ORs, multivariable modeling was limited by the number of events. For postoperative delirium, a parsimonious multivariable model adjusted for age ≥65 years and sex was used; however, additional adjustment for cognitive status, medication exposure, operative time, nutrition, sarcopenia, and adjuvant therapy was not feasible because these variables were unavailable or incomplete. Third, mFI-5 does not include nutrition, sarcopenia, cognition, inflammatory status, or social vulnerability, all of which may be important in OSCC. Fourth, standardized preoperative cognitive screening and scheduled postoperative delirium screening tools were not routinely used throughout the study period; therefore, hypoactive delirium may have been missed. Fifth, sedative, opioid, anticholinergic, and benzodiazepine exposures were not consistently available for analysis. Sixth, complete information on adjuvant therapy was not available for the whole cohort, limiting interpretation of OS and DFS. Seventh, the mFI-5 captures the presence but not the severity or degree of control of its component conditions; detailed measures such as glycemic control, blood pressure control, end-organ damage, and heart failure severity were not uniformly available. Finally, this was a single-center study, and the results may not be generalizable to institutions with different surgical indications, HCU admission criteria, reconstruction protocols, and delirium prevention programs.

From a practical standpoint, the present results support a two-step approach to perioperative risk assessment. First, the mFI-5 can be used as a rapid screening instrument because it can be calculated from routinely available clinical information without additional patient burden. Second, patients identified as frail according to the mFI-5 should undergo a more detailed evaluation focused on potentially modifiable factors, including nutrition, skeletal muscle mass, medication burden, alcohol and smoking history, sleep disturbance, and cognitive vulnerability. This approach may be particularly valuable in OSCC, where complex reconstructive procedures and postoperative communication difficulties may increase delirium risk. Even when mFI-5 is not independently predictive of survival, identifying delirium-prone patients remains clinically relevant because delirium prevention is actionable and may improve recovery trajectories.

Moreover, because the diagnosis of delirium was based on clinical documentation consistent with DSM-5 criteria rather than scheduled screening with CAM, CAM-ICU, ICDSC, or Nu-DESC, hypoactive delirium may have been under-recognized. This may have resulted in an underestimation of delirium incidence and should be considered when comparing the present results with studies that used standardized daily screening instruments.

Despite these limitations, the study provides clinically useful information. The findings support the use of mFI-5 as a practical screening tool that can be calculated easily from routine preoperative records. However, the index should be used to identify patients requiring further assessment rather than to make definitive predictions. In patients with OSCC, particularly those undergoing extensive surgery, mFI-5-defined frailty may help clinicians recognize delirium vulnerability and implement preventive perioperative care. Future multicenter studies with larger cohorts, standardized delirium assessment, complete treatment data, and comprehensive geriatric and nutritional evaluation are required to determine whether combined models can improve risk prediction and patient-centered outcomes.

## 5. Conclusions

mFI-5-defined frailty was associated with postoperative delirium but not with OS or DFS in patients undergoing surgical treatment for OSCC. In a parsimonious multivariable model adjusted for age ≥65 years and sex, mFI-5-defined frailty remained significantly associated with postoperative delirium. Nevertheless, given the retrospective design, limited sample size, and potential residual confounding, the findings should be interpreted as an association rather than definitive evidence of independent prediction. The mFI-5 is a convenient screening tool, but it does not capture the multidimensional nature of frailty in OSCC and, in this cohort, was largely driven by diabetes mellitus and hypertension rather than functional dependence. Comprehensive assessment incorporating comorbidities, nutritional status, sarcopenia, cognition, tumor burden, treatment factors, and medication exposure may improve perioperative risk stratification and delirium prevention.

## Figures and Tables

**Table 1:** Patient Characteristics and Frailty Status.

Variable	Category	Non-Frail (*n* = 106)	Frail (*n* = 21)	*p*-Value
Age (years)	<65	41 (38.7)	7 (33.3)	0.644
	≥65	65 (61.3)	14 (66.7)	
Sex	Male	63 (59.4)	17 (81.0)	0.062
	Female	43 (40.6)	4 (19.0)	
Smoking history	Yes	44 (41.5)	11 (52.4)	0.358
	No	62 (58.5)	10 (47.6)	
Alcohol consumption	Yes	43 (40.6)	10 (47.6)	0.549
	No	63 (59.4)	11 (52.4)	
Performance status	0	95 (89.6)	17 (81.0)	0.066
	1	11 (10.4)	3 (14.3)	
	2	0 (0.0)	1 (4.8)	
ASA classification	1	8 (7.5)	0 (0.0)	0.313
	2	41 (38.7)	8 (38.1)	
	3	28 (26.4)	8 (38.1)	
	Unknown	29 (27.4)	5 (23.8)	
Primary site	Tongue	46 (43.4)	9 (42.9)	0.912
	Mandibular gingiva	36 (34.0)	9 (42.9)	
	Buccal mucosa	9 (8.5)	1 (4.8)	
	Maxillary gingiva	8 (7.5)	1 (4.8)	
	Floor of mouth	7 (6.6)	1 (4.8)	
Stage classification	I	2 (1.9)	0 (0.0)	0.806
	II	20 (18.9)	3 (14.3)	
	III	12 (11.3)	3 (14.3)	
	IVA	60 (56.6)	11 (52.4)	
	IVB	12 (11.3)	4 (19.0)	
M classification	M0	106 (100.0)	21 (100.0)	--
T classification	T1	2 (1.9)	0 (0.0)	0.675
	T2	29 (27.4)	5 (23.8)	
	T3	14 (13.2)	4 (19.0)	
	T4a	50 (47.2)	8 (38.1)	
	T4b	11 (10.4)	4 (19.0)	
N classification	N0	56 (52.8)	8 (38.1)	0.661
	N1	14 (13.2)	5 (23.8)	
	N2b	31 (29.2)	7 (33.3)	
	N2c	4 (3.8)	1 (4.8)	
	N3b	1 (0.9)	0 (0.0)	
Flap type	Anterolateral thigh flap	33 (31.1)	5 (23.8)	0.779
	Rectus abdominis flap	18 (17.0)	6 (28.6)	
	Fibula flap	26 (24.5)	5 (23.8)	
	Others	4 (3.8)	1 (4.8)	
	None	25 (23.6)	4 (19.0)	
Reoperation of free flap	Yes	3 (3.9)	2 (12.5)	0.170
(*n* = 93)	No	74 (96.1)	14 (87.5)	
Postoperative pneumonia	Yes	20 (18.9)	4 (19.0)	0.985
	No	86 (81.1)	17 (81.0)	
Postoperative delirium	Yes	21 (19.8)	9 (42.9)	0.023 *
	No	85 (80.2)	12 (57.1)	
Surgical site infection	Yes	12 (11.3)	2 (9.5)	0.810
	No	94 (88.7)	19 (90.5)	

* *p* < 0.05. Percentages for free-flap reoperation were calculated among patients who underwent free-flap reconstruction. M0 indicates no documented distant metastasis at surgery.

**Table 2:** Univariable logistic regression analysis for postoperative complications according to mFI-5-defined frailty.

Outcome	Population Analyzed	OR	95% CI	*p*-Value
Postoperative pneumonia	All patients	1.01	0.27–3.10	0.985
Postoperative delirium	All patients	3.04	1.11–8.16	0.031
Surgical site infection	All patients	0.83	0.12–3.36	0.810
Free-flap reoperation	Free-flap patients only	3.48	0.53–22.74	0.170

ORs represent the odds of each postoperative complication in frail patients compared with non-frail patients. *p*-values were obtained from univariable logistic regression models. CI, confidence interval; OR, odds ratio.

**Table 3:** Multivariable Logistic Regression Analysis for Postoperative Delirium.

Variable	Adjusted OR	95% CI	*p*-Value
mFI-5-defined frailty (reference: non-frail)	3.07	1.08–8.60	0.035
Age ≥ 65 years (reference: <65 years)	2.37	0.95–6.57	0.066
Female sex (reference: male)	1.09	0.44–2.67	0.849

The reference categories were non-frail, age < 65 years, and male sex. CI, confidence interval; OR, odds ratio.

**Table 4:** The Distribution of the Modified 5-Item Frailty Index in Patients.

mFI-5 Score	*n*	Diabetes Mellitus	Hypertension Requiring Medication	Respiratory Disease (COPD, Pneumonia)	Congestive Heart Failure	Functional Status
0	52	0	0	0	0	0
1	54	10	42	2	0	0
2	20	19	20	0	1	0
3	1	1	1	1	0	0

## Data Availability

The data presented in this study are available on reasonable request from the corresponding author. The data are not publicly available due to privacy and ethical restrictions concerning patient information.

## Abbreviations

The following abbreviations are used in this manuscript:

ACS-NSQIP	American College of Surgeons National Surgical Quality Improvement Program
ASA	American Society of Anesthesiologists
CDC	Centers for Disease Control and Prevention
COPD	Chronic obstructive pulmonary disease
DFS	Disease-free survival
DSM-5	Diagnostic and Statistical Manual of Mental Disorders, Fifth Edition
HCU	High care unit
HNC	Head and neck cancer
mFI-5	Modified 5-item frailty index
OS	Overall survival
OSCC	Oral squamous cell carcinoma
PS	Performance status
SSI	Surgical site infection
TNM	Tumor–node–metastasis classification
UICC	Union for International Cancer Control
